# Supplementary material for: Lateral advection supports nitrogen export in the oligotrophic open-ocean Gulf of Mexico
Source: Nat Commun. 2021 Jun 3;12:3325. doi: 10.1038/s41467-021-23678-9 (PMC8175579; doi:10.1038/s41467-021-23678-9)
Supplement: Supplementary file 1 — Supplemental Figures and Discussion [file 41467_2021_23678_MOESM1_ESM.pdf]

Supplemental for Lateral Advection Supports Nitrogen Export in the Oligotrophic Open-Ocean Gulf of Mexico

Thomas B. Kelly[1,2,*,†], Angela N. Knapp[1], Michael R. Landry[3], Karen E. Selph[4], Taylor A. Shropshire[1,2], Rachel K. Thomas[1], and Michael R. Stukel[1,2]

[1] Department of Earth, Ocean and Atmospheric Sciences, Florida State University, Tallahassee, FL, USA

[2] Center for Ocean-Atmospheric Prediction Studies, Florida State University, Tallahassee, FL, USA

[3] Integrative Oceanography Division, Scripps Institution of Oceanography, La Jolla, CA, USA

[4] Department of Oceanography, University of Hawaii at Manoa, Honolulu, HI, USA

[†] Presently at: College of Fisheries and Ocean Science, University of Alaska Fairbanks, Fairbanks, AK, USA

* Correspondence to Thomas B Kelly (tbkelly@alaska.edu)

Supplemental Figures

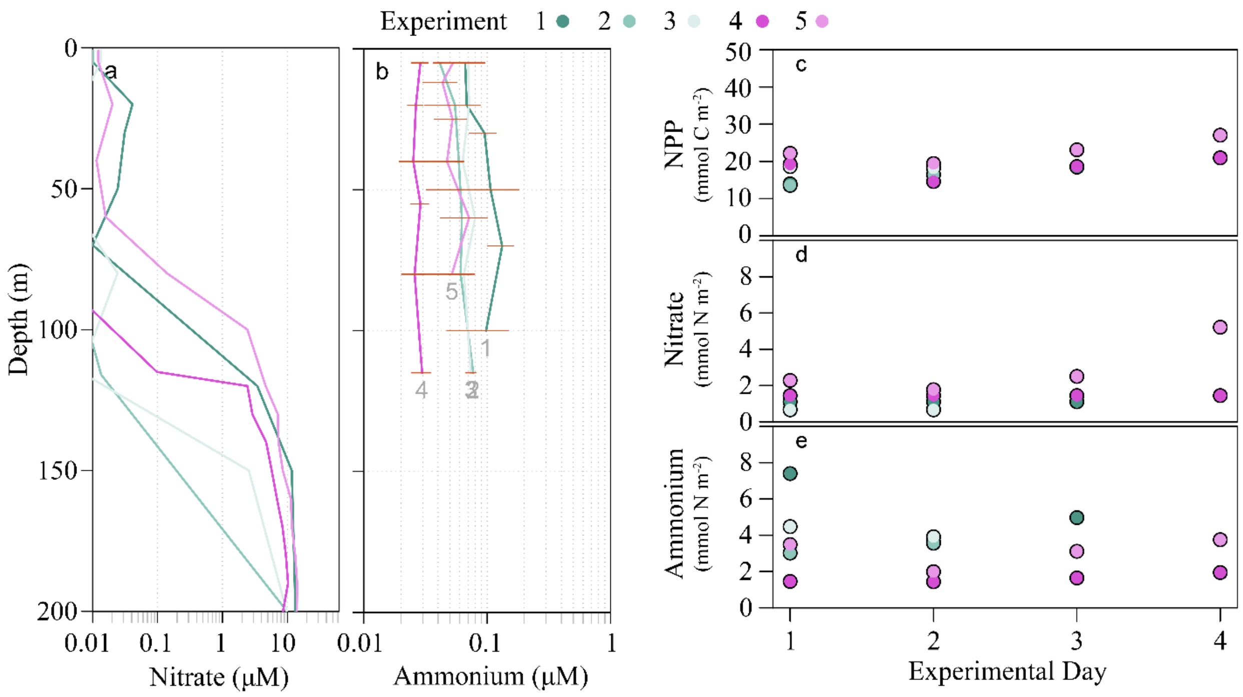

Supplemental Figure 1. Vertical nutrient profiles and daily upper euphotic zone rates and inventories. (a-b) Nitrate and ammonium profiles averaged by Lagrangian experiment as indicated by color. (c-e) Timeseries of UEZ-integrated net primary productivity and inventories of nitrate and ammonium for each day of an experiment. Error bars correspond to 1 SD of the measurements.

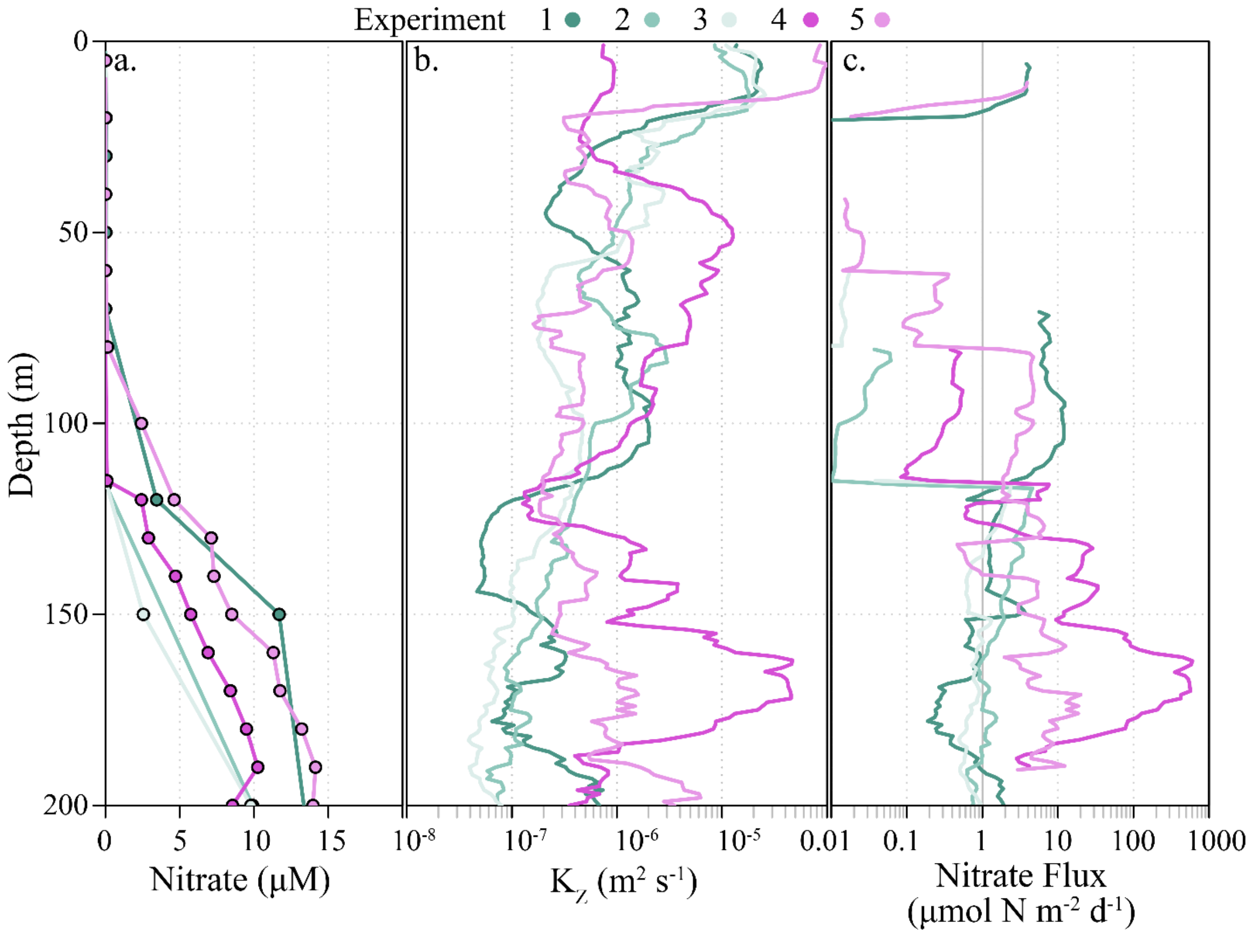

Supplemental Figure 2. Calculation of vertical mixing of nitrate. (a) Vertical profiles of nitrate concentrations, (b) vertical eddy diffusivity, and (c) calculated nitrate flux. Values shown were averaged for each Lagrangian experiment and color coded as indicated.

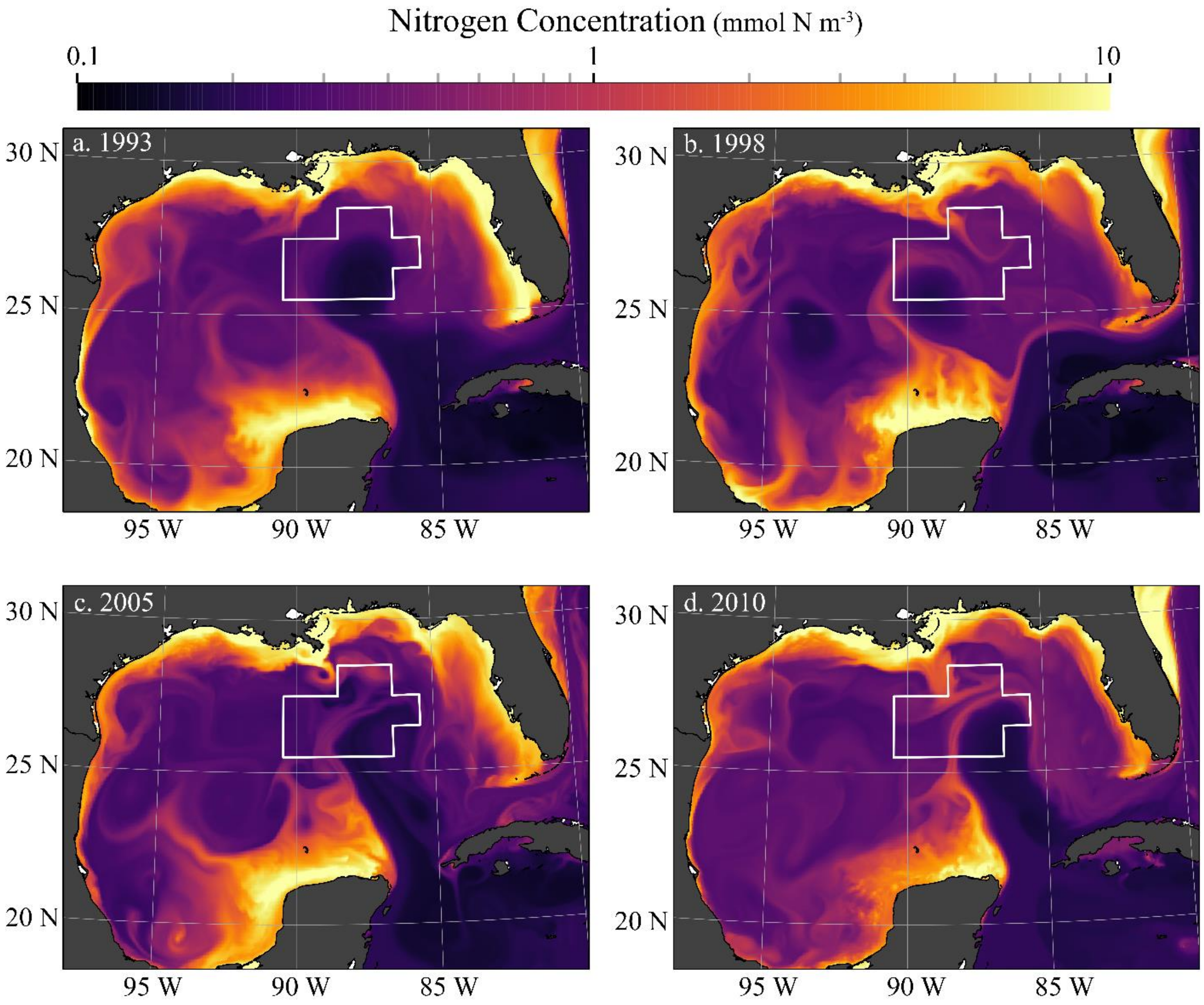

Supplemental Figure 3. Map of GoM with upper euphotic zone total nitrogen concentrations (NEMURO-GOM) for four representative mays. Data are monthly averages for each respective year for depths 0 – 55 m depth and include all nitrogen containing state variables.

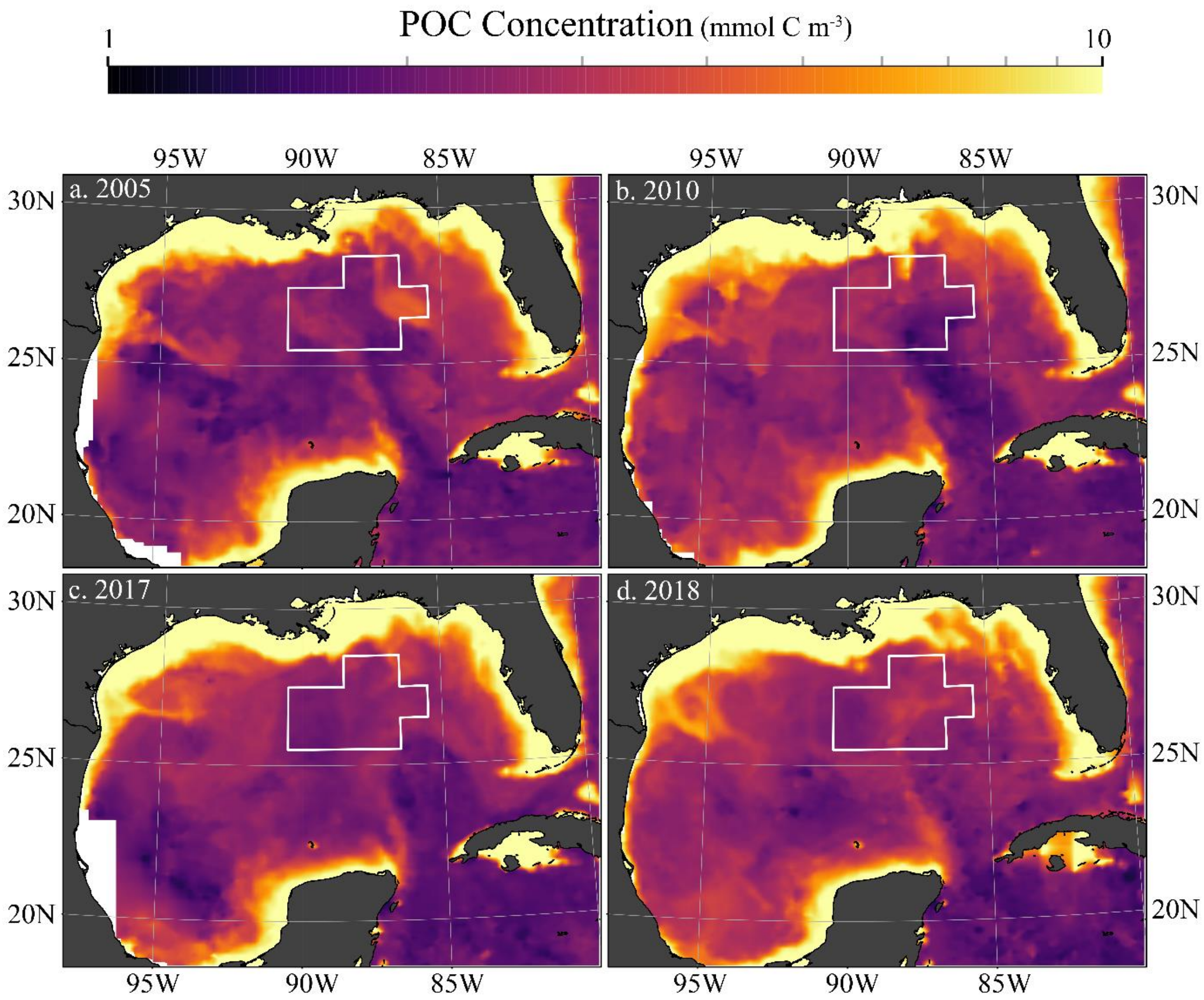

Supplemental Figure 4. Map of Gulf of Mexico with surface particulate organic carbon inventories (via MODIS) for two representative mays (a-b) and the two observed mays (c-d). Data are 8-day composites for mid-May with 8 km lateral resolution. Years are as indicated.

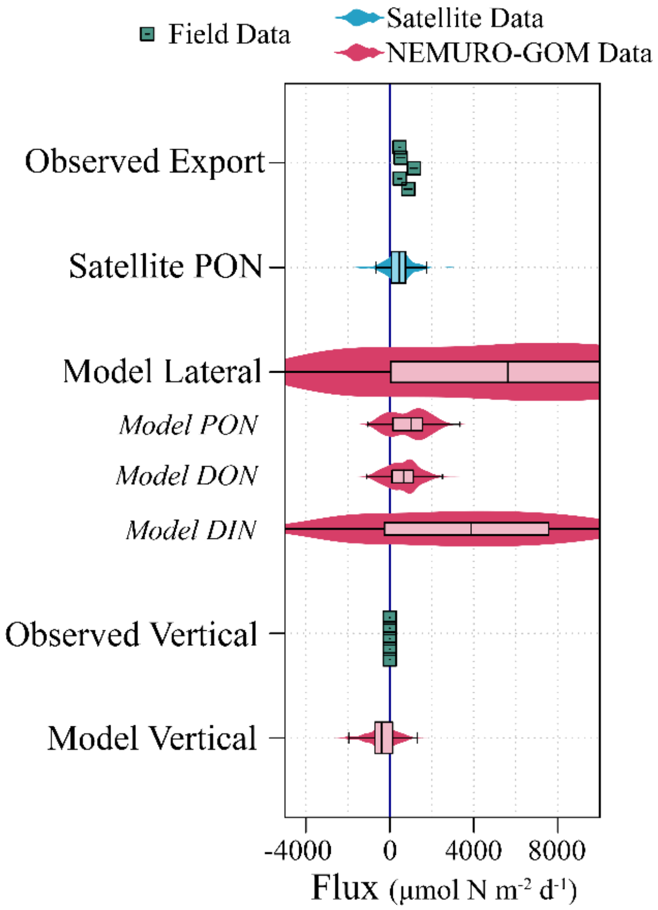

Supplemental Figure 5. Comparison of lateral nitrogen supply to observed export production for entire euphotic zone (upper + lower euphotic zone; UEZ + LEZ). Observed export was at the base of the euphotic zone. Satellite lateral particulate organic nitrogen and NEURO-GOM fluxes were calculated as in Figure 3. NEMURO-GOM vertical fluxes are integrated to 135 m and include upwelling and turbulent mixing but not export by sinking particles. Positive flux values indicate net input into the integration volume. Flux values are normalized to lateral area. Boxes were determined from the 25th, 50th, and 75th percentile of the data, with whiskers indicating the limits of data extending 1.5x the interquartile range of the data.

Supplemental Discussion

Constraining the Source of Laterally Transported Organic Matter

In this study, we investigated lateral advection using a hydrodynamic model and remote sensing data products and found a significant association between mesoscale circulation and large-scale transport into the central GoM study region. Furthermore, estimated net lateral transport appears to balance observed nitrogen export—an export term otherwise unbalanced by *in situ* processes. Yet, without additional validation, the remote sensing and model products should not be used to constraint the composition of the source material from which the laterally transported nitrogen is derived. Whether this bioavailable nitrogen is sourced from (1) subsurface nitrate, (2) N_2-fixation, or (3) terrestrial sources remains unresolved, yet several patterns are evident through this study and others.

Geographically, the source regions of the laterally advected nitrogen can be broadly identified based on a mean state approximation (Supplemental Figures 3-4). A substantial proportion of lateral transport is carried through the southern boundary of the control box, which is likely derived from entrainment and localized upwelling associated with the interaction of the Campeche Bank and Loop Current[1,2] (Figure 4, Supplemental Figures 3-4).

Applying an eddy detection algorithm[3] to NEMURO-GOM, net fluxes associated with eddies were small (mean: 44 µmol N m^{-2} d^{-1}) relative to average net flux (1165 µmol N m^{-2} d^{-1}). This, however, is likely due to our criteria for an eddy, which implies no lateral divergence (i.e. closed stream function) and thus excludes phenomenon such as filaments and jets that often form on the edges of eddies[4]. Although mesoscale eddies, which are shed by the Loop Current[5,6], only carry a small proportion (~4%) of the net lateral transport, they likely force surrounding flow fields[7] that may contribute significant flux over short durations (compared to the Loop Current). Nevertheless, additional data are necessary to determine both the delivery mechanisms and sourcing mechanisms responsible for the lateral N transport in the GoM.

Nitrogen isotopic signatures (i.e. $\delta^{15}N$) carry with them information about their original sources (e.g. subsurface nitrate: 2 – 4 ‰ vs N_2-fixation: -2 – 0 ‰), albeit continuously modified by biological processes[8]. The mass-balance constraints for nitrogen and ^{15}N, as presented, are consistent with upwelled, laterally sourced nitrate[9] and the conclusion that N_2-fixation is not substantial in these oligotrophic waters[10]. However, it is not possible to positively associate export material (3 – 5‰) with a unique combination of end-members due to the wide range of isotopic signatures for riverine (6 – 8‰)[11], atmospheric (-5 – 4‰)[12], and biotic (-2 – 0‰) sources of nitrogen, especially for end members with small relative contributions (such as atmospheric deposition in the GoM[9]). However, without a significant

source of sufficiently low $\delta^{15}N$ (i.e. << 3‰), N from the northern GoM margin is unlikely be a significant source of organic matter to the oligotrophic GoM. Finally, previous studies have come to mixed conclusions on the degree of connectivity between the shelf environment and the pelagic GoM [2,6,13]. Combined with the present questions regarding the source of the laterally transported N, process studies in the pelagic GoM are necessary to thoroughly investigate these shelf-basin interactions.

Implications for Vertical Connectivity

Given that the LEU was disproportionally responsible for nitrate uptake[14], although supporting only 21 – 38% of NPP, one might conclude that export production is centered within the LEZ and that export production may thus be supported by episodic fluxes of nitrate at depth. However, observations of the export flux strongly refute such a hypothesis. Particle flux out of the UEZ exceeded that of the LEZ implying that the LEZ as a zone of net remineralization and not of particle formation. This vertical partitioning of export production illustrates the potential problems of including the entire euphotic zone into a single mass-budget. Performing an identical mass-budget for the entire euphotic zone slightly reduces the substantial mismatch determined between local *in situ* nitrogen sources and sinks by averaging across areas of particle production and heterotrophic consumption. However, importantly, it does not change the overall conclusions regarding the role of lateral transport (Supplemental Figure 5). Indeed, vertical integrations of lateral flux become increasingly sensitive to depth due to the large inventory of subsurface nitrate and spatial variability in euphotic zone depth. Integrating NEMURO-GOM to 135 m (comparable to the depth of most sediment trap deployments) does not significantly change lateral PON, DON or vertical flux values (Figure 3) but does substantially augment lateral DIN fluxes. By restricting our primary analysis to the UEZ, we not only get a clearer picture of euphotic zone nitrogen requirements (e.g. export flux at 60 m) but also mitigate sensitivities associate with depth integrations in regions of large vertical gradients.

Supplemental References

1. Merino, M. Upwelling on the Yucatan Shelf: hydrographic evidence. *J. Mar. Syst.* **13**, 101–121 (1997).
2. Otis, D., Le Hénaff, M., Kourafalou, V., McEachron, L. & Muller-Karger, F. Mississippi River and Campeche Bank (Gulf of Mexico) episodes of cross-shelf export of coastal waters observed with satellites. *Remote Sens.* **11**, 723 (2019).
3. Laxenaire, R. *et al.* Anticyclonic eddies connecting the western boundaries of Indian and Atlantic Oceans. *J. Geophys. Res. Oceans* **123**, 7651–7677 (2018).
4. Nagai, T. *et al.* Dominant role of eddies and filaments in the offshore transport of carbon and nutrients in the California Current System. *J. Geophys. Res. Oceans* **120**, 5318–5341 (2015).
5. Oey, L.-Y., Ezer, T. & Lee, H.-C. Loop current, rings and related circulation in the Gulf of Mexico: A review of numerical models and future challenges. in *Geophysical Monograph Series* (eds. Sturges, W. & Lugo-Fernandez, A.) 31–56 (2013). doi:10.1029/161GM04.
6. Zhong, Y. & Bracco, A. Submesoscale impacts on horizontal and vertical transport in the Gulf of Mexico: Submesoscale transport in Gulf of Mexico. *J. Geophys. Res. Oceans* **118**, 5651–5668 (2013).
7. Sahl, L. E., Wiesenburg, D. A. & Merrell, W. J. Interactions of mesoscale features with Texas shelf and slope waters. *Cont. Shelf Res.* **17**, 117–136 (1997).
8. Casciotti, K. L. Nitrogen and oxygen isotopic studies of the marine nitrogen cycle. *Annu. Rev. Mar. Sci.* **8**, 379–407 (2016).
9. Howe, S., Miranda, C., Hayes, C. T., Letscher, R. T. & Knapp, A. N. The dual isotopic composition of nitrate in the Gulf of Mexico and Florida Straits. *J. Geophys. Res. Oceans* **125**, (2020).
10. Knapp, A. N. *et al.* Constraining the sources of nitrogen fueling phytoplankton and food webs in the Gulf of Mexico using nitrogen isotope budgets. *J. Plankton Res.* (submitted).
11. BryantMason, A., Xu, Y. J. & Altabet, M. Isotopic signature of nitrate in river waters of the lower Mississippi and its distributary, the Atchafalaya. *Hydrol. Process.* **27**, 2840–2850 (2013).
12. Dillon, K. S. & Chanton, J. P. Nutrient transformations between rainfall and stormwater runoff in an urbanized coastal environment: Sarasota Bay, Florida. *Limnol. Oceanogr.* **50**, 62–69 (2005).

13. Barkan, R. *et al.* Submesoscale dynamics in the northern Gulf of Mexico. Part II: Temperature–salinity relations and cross-shelf transport processes. *J. Phys. Oceanogr.* **47**, 2347–2360 (2017).

14. Yingling, N. *et al.* Taxon-specific phytoplankton growth, nutrient limitation, and light limitation in the oligotrophic Gulf of Mexico. *J. Plankton Res.* (in press).
